# Effect of Cinnamaldehyde Addition on Injectable Gypsum–Calcium Carbonate Hydrogel Paste with Ultraviolet Light Polymerization: Bone Scaffold Material for Implant

**DOI:** 10.1055/s-0045-1807729

**Published:** 2025-05-02

**Authors:** Anne Handrini Dewi, Muhammad Akhsan Pridatama, Dena Kusuma Arum, Mas Sahidayana Mohktar, Hersandy Dayu Kusuma, Andi Triawan

**Affiliations:** 1 Department of Dental Biomedical Sciences, Faculty of Dentistry, Universitas Gadjah Mada, Yogyakarta, Indonesia; 2 Department of Biomedical Engineering, Faculty of Engineering, Universiti Malaya, Kuala Lumpur, Malaysia; 3 Department of Chemistry, Faculty of Mathematics and Natural Sciences, Universitas Padjadjaran, Sumedang, Indonesia; 4 Department of Orthodontics, Faculty of Dentistry, Universitas Gadjah Mada, Yogyakarta, Indonesia

**Keywords:** cinnamaldehyde, injectable paste, ultraviolet, polymerization, scaffold, implant

## Abstract

**Objective:**

Hydrogel-based, gypsum, calcium carbonate (CaCO _3_ ) bone scaffolding materials and antibacterial extracts from cinnamaldehyde herbal ingredients are a combination of smart materials that are abundant and environmental friendly. That component is a promising candidate for bone scaffold material. This prototype has been designed as an injectable paste that is easy to apply, fills in bone and dental defects, and quickly polymerizes with the help of ultraviolet (UV) light. The purpose of this study is to investigate the effects of adding cinnamaldehyde to injectable gypsum–CaCO _3_ hydrogel paste that has undergone UV light polymerization for biodegradable implant material.

**Materials and Methods:**

A composite material was synthesized named Cia by a combination of gelatin, gypsum, CaCO _3_ , and cinnamaldehyde compound assisted by UV light polymerization. An *in vitro* and *in vivo* quasi-experiments were conducted in this study, including material characterization and testing. Material characterization was performed using Fourier transform infrared spectroscopy) and scanning electron microscope. Material testing examined the swelling ratio and degradation rate. Antibacterial activity was performed as *in vitro* testing undergone *Streptococcus sanguinis* and *Pseudomonas aeruginosa* . Data were analyzed statistically using an independent *t* -test ( *p* < 0.05). A total of 21 male Wistar rats were used *in vivo* study. A femoral condyle was chosen as a hard tissue representative of the jaw. Tissues were then stained with hematoxylin–eosin and Mallory staining observed under a light microscope to identify the tissue regeneration and implant remaining.

**Conclusion:**

Synthesized material that is added by cinnamaldehyde could be an implant material for inducing tissue regeneration.

## Introduction

Hydrogels have gained significant attention in biomedical applications due to their biocompatibility, 1 ability to mimic the extracellular matrix, 2 and potential as scaffolds for tissue regeneration. 3 4 5 In bone and tissue engineering, hydrogels have been widely explored as injectable scaffolds that conform to irregular bone defects and facilitate bone regeneration. Injectable hydrogels are particularly advantageous due to their minimally invasive application, ease of use, and ability to deliver bioactive molecules or therapeutic agents directly to the injury. 6 Injectable hydrogels are particularly advantageous due to their minimally invasive application, ease of use, and ability to deliver bioactive molecules or therapeutic agents directly to the injury site. 7 Injectable hydrogels offer numerous advantages, such as their minimally invasive application, user-friendly characteristics, and the capacity to deliver bioactive molecules or pharmacological agents directly to the injury site. 8 Hydrogels based on calcium carbonate (CaCO _3_ ) have particularly attracted scholarly interest due to their biodegradability, bioactivity, and function in serving as a reservoir for calcium ions, which are essential for the process of bone regeneration. 8 Among hydrogel-based materials, CaCO _3_ -based hydrogels have been of particular interest due to their biodegradability, bioactivity, and ability to serve as a reservoir for calcium ions, which are essential for bone mineralization and regeneration. 9 10 Gypsum has been widely employed in bone defect filling due to its osteoconductive properties and biocompatibility. 11 12 However, despite their benefits, gypsum-based materials often suffer from poor mechanical strength and rapid degradation, limiting their clinical application. To address these limitations, this study has explored the incorporation of CaCO _3_ within hydrogel systems, enhancing the mechanical properties while maintaining osteoconductivity. Furthermore, ultraviolet (UV) light polymerization has emerged as a promising technique for improving the structural integrity of hydrogel scaffolds. 13 UV-induced cross-linking strengthens the hydrogel network, providing better mechanical stability and prolonged degradation time, which are essential for bone scaffolds. 14

In addition to mechanical reinforcement, incorporating bioactive compounds can further improve hydrogel-based materials by enhancing biological activity. 15 Cinnamaldehyde, a natural compound derived from cinnamon bark, has demonstrated 15 anti-inflammatory and antioxidant properties. 16 17 These properties make cinnamaldehyde an attractive additive for biomaterials used in tissue engineering, particularly in preventing infections and promoting wound healing. 18 In the context of bone regeneration, cinnamaldehyde has the potential to enhance the performance of hydrogel scaffolds by providing additional protection against bacterial contamination and promoting a favorable environment for bone cell proliferation. 19 20 UV-light utilization for the cross-linking of the hydrogel enhances the material's mechanical integrity, whereas cinnamaldehyde provides bioactive properties that may reduce the risk of infection and accelerate the healing process. 17 Furthermore, its controlled release from UV-polymerized hydrogels could ensure sustained antimicrobial activity over time, reducing postimplantation complications. 21

Despite its promising bioactive properties, the effect of cinnamaldehyde on gypsum–CaCO _3_ -based hydrogels, particularly those using UV polymerization, remains underexplored. Integrating cinnamaldehyde with UV-polymerized hydrogels presents a novel approach for enhancing both the mechanical and biological properties of injectable bone scaffolds. This study aims to investigate the influence of cinnamaldehyde on the mechanical strength, antimicrobial activity, and osteoconductive potential of injectable gypsum–CaCO _3_ hydrogel paste polymerized with UV light. By evaluating these characteristics, this research provides insights into the development of advanced biomaterials for bone tissue engineering and proposes an innovative strategy for improving the performance of injectable hydrogels in clinical applications.

## Materials and Methods

### Precursors

Precursor materials such as gypsum (calcium sulfate [CaSO _4_ ]), CaCO _3_ , and sodium citrate were purchased from Wako Pure Chemical Industries Ltd. (Osaka, Japan). Type B gelatin was provided by Nitta Gelatin Inc. (Osaka, Japan), while cinnamaldehyde was purchased from Merck (Darmstadt, Germany). All other chemicals used were of pure analytical grade.

### Material Preparation

Sodium citrate 0.125 g is added to aquadest 5 mL by stirring until dissolved, and then 0.415 g CaCO _3_ and 0.05 g CaSO _4_ are added to the solution by continuous stirring until homogeneous for 5 minutes. Subsequently, 1 g gelatin and 0.1 mL cinnamaldehyde at varying concentrations are added to the solution mixture until homogeneous for 15 minutes at a temperature of 40°C. After that, 0.1 g hydroxy ethyl cellulose and 0.272 g camphorquinone are added to the solution mixture and stirred for 5 minutes until the solution becomes viscous. Finally, the solution was molded into a mold container, and the sample was dried under UV irradiation for 20 seconds and left at room temperature. For the study, completely set and dried cylindrical samples with a diameter of 6 mm and a thickness of 3 mm were used, and the sample was named Cia. The Cia-1, Cia-2, and Cia-3 are synthesized materials with the addition of 1, 2, and 3% cinnamaldehyde, respectively.

### Material Characterization

Characterization of the synthesized materials was conducted using Fourier transform infrared spectroscopy (FTIR) on the Thermo Scientific Nicolet iS10 apparatus to determine the existing functional groups. Additionally, the particle morphology of the materials was investigated utilizing a scanning electron microscope (SEM) coupled with energy dispersive X-ray spectroscopy, specifically the JEOL JSM-6510LA instrument, operating at a voltage of 15 kV.

### Material Testing

#### Swelling Test

The Cia samples were immersed in phosphate-buffered saline (PBS) at 37°C, pH 7.4 for the water sorption capacity test. The Cia materials were initial weight ( *W*_0_ ) before immersion, and the swollen samples were investigated at a determined interval time ( *W*_t_ ) until the degradation process began. The swelling ratio of the scaffolds ( *W*_sw_ ) was calculated using Eq. (1) . The values were expressed as the mean ± standard deviation:



#### Degradability Test

For the degradability test, simulated body fluid (SBF) at pH 7.4 and 37°C was used as a solution for immersing cylindrical specimens of Cia materials for 1, 2, 4, and 8 weeks. The sizes of Cia materials were controlled within the diameter of 6 mm and a thickness of 3 mm. There were three specimens tested for each experimental condition. The reagent's grades such as NaHCO _3_ , KCl, MgCl _2_ .6H _2_ O, CaCl _2_ , Na _2_ SO _4_ , NaCl, and K _2_ HPO _4_ .3H _2_ O were dissolved into deionized water as the SBF solution. The ionic concentration of SBF (mM) was according to the description. The weight of the dried discs was determined and marked as *W*_0_ . After the designated soaking duration, the specimens were cautiously extracted from the SBF and subsequently wiped with filter paper to support the drying process. Following the attainment of a dry condition, the specimens were reweighed, and their mass was noted as *W*_1_ . The rate of weight loss ( *L* ) was calculated according to Eq. 2 presented below:



### *In vitro* Study

In this study, two facultatively anaerobic bacterial cultures were used: *Pseudomonas aeruginosa* ATCC 10145, a gram-negative, and *Streptococcus* *sanguinis* ATCC 10556, a gram-positive. Following an overnight incubation period, a sterile cotton swab was used to apply a bacterial suspension (1.5 × 108 CFU/mL) to culture plates containing brain heart infusion agar to achieve consistent microbial growth. The medium was brought into close contact with cylindrical discs containing different concentrations of cinnamon aldehyde. A standard disc containing 5 mg of ciprofloxacin as the positive control and a disc containing phosphate buffer solution (PBS) as the negative control were also included in the test agar plate for comparison. For 24 hours, the agar plates were incubated at 37°C. Antibacterial activity was assessed by measuring the clear activity zones.

The medium was brought into close contact with cylindrical discs containing different concentrations of cinnamon aldehyde. For comparison, the test agar plate also contained a standard disc with 5 mg of ciprofloxacin as the positive control and a disc with PBS as the negative control. For 24 hours, the agar plates were incubated at 37°C. Antibacterial activity was assessed by measuring the clear activity zones' diameters. Three duplicates of each study were conducted, and the mean and standard deviation were computed. Unless otherwise noted in the test, all reagents were acquired from Sigma Aldrich (Darmstadt, Germany).

### *In vivo* study

The Ethics Committee of the Faculty of Dentistry, Universitas Gadjah Mada, accepted the *in vivo* study that involved animal experimentation (number 76/UNI/KEP/FKG-RSGM/EC/2023). To acclimate to the daily feed, water, and 12-hour/12-hour light/dark cycle, 21 Wistar rats, male, 4 months old, and weighing between 250 and 350 g, were housed in an animal care facility for 7 days. Throughout the investigation, national rules for the use and care of laboratory animals were followed. The animals will be kept in cages at Universitas Gadjah Mada's Integrated Research and Testing Laboratory (LPPT). Sterile gauze dipped in betadine was used to clean and shave the surgical site. Xylasin (0.55–1.1 mg/kg body weight) and ketamine (11–22 mg/kg body weight) were injected intramuscularly into the animal while it was under general anesthesia. Subcutaneous and femoral condyle implantation are the two surgical techniques used to evaluate the implant's soft and hard tissue response.

*Subcutaneous implantation* : Using a dorsal midline incision that was 1 cm long on both sides of the spinal column, cylindrical implants were inserted into a subcutaneous pouch. Resorbable 2.0 Vicryl suture (Ethicon, Johnson & Johnson Indonesia, Jakarta, Indonesia) was used to seal the skin. On days 3, 7, and 14 after implantation, the rats were killed by an overdose of xylazine and ketamine. Following that, the implant and the surrounding tissue were removed right away for additional histological processing. The sections were stained with hematoxcylin–eosin (HE) to show the inflammatory cells around the material. Histological investigation was performed with an Olympus stereo microscope in Japan and viewed at ×32 magnification.

*Femoral condyle implantation* : A 1-cm longitudinal incision was made to reveal the lateral side of the right femoral condyle ( *n* = 3). The drilled hole was then subsequently widened to be 2 mm wide and 3 mm deep to reach the final defect diameter of 2.5 mm. Low rotational drill rates and constant internal cooling with saline solution were used to prepare the defects. Each rat's bone defect was filled with a cylindrical implant that measured 2.5 mm in width and 3 mm in length. Following implant placement, Vicryl 2.0 (Ethicon, Johnson & Johnson Indonesia) suture material was used to close the muscles and skin in distinct layers. For 5 days following surgery, the rats were given 10 mL/20 to 40 kg intramuscular doses of the antibiotic Interflox-100 (Interchemix, Horsterweg 26, Maastricht, the Netherlands) to lower the risk of perioperative infection. Every day, the postoperative state, including the surgical wound, food consumption, level of activity, and clinical indications of infection was observed. The implantation times were 14 and 30 days. At the end of their respective implantation times, rats were killed by ketamine overdose and stored in histology preparation for histological procedures.

#### Histology Preparation for Hard Tissue Implantation

The femoral condyles were then extracted, and any extra tissue was cut out right away. A diamond bur was used to cut the condyles into smaller samples in specific implant locations, and they were then fixed for a day in a 10% buffered formalin solution. After that, 10% ethylenediaminetetraacetic acid (Titriplex III, Merck, Darmstadt, Germany) was used to decalcify the specimens. For a month, the solution was shaken twice a day and replaced twice a week. Following decalcification, the bone samples were embedded in paraffin and dehydrated in a succession of ethanol grades ranging from 50 to 100%. Perpendicular to the specimen's longitudinal axis, 5 µm cross-sectional serial slices were created. HE was used to stain sections to examine the scatter of inflammatory cells and bone formation and Mallory staining was used to measure the density of collagen fibers. Histological investigation was performed with an Olympus stereo microscope in Japan. Every section of subcutaneous and femoral condyle implantation was examined using a description and viewed at ×32 magnification.

### Statistical Analysis

In this study, the primary statistical technique used was a Student's *t* -test to assess the presence of significant differences within the data. This approach was selected because it was appropriate for the study's design and the type of data. By computing analysis results with a significance level ( *a* ) set at 95%, which corresponds to a *p* -value of 0.05.

## Results

**Fig. 1:**
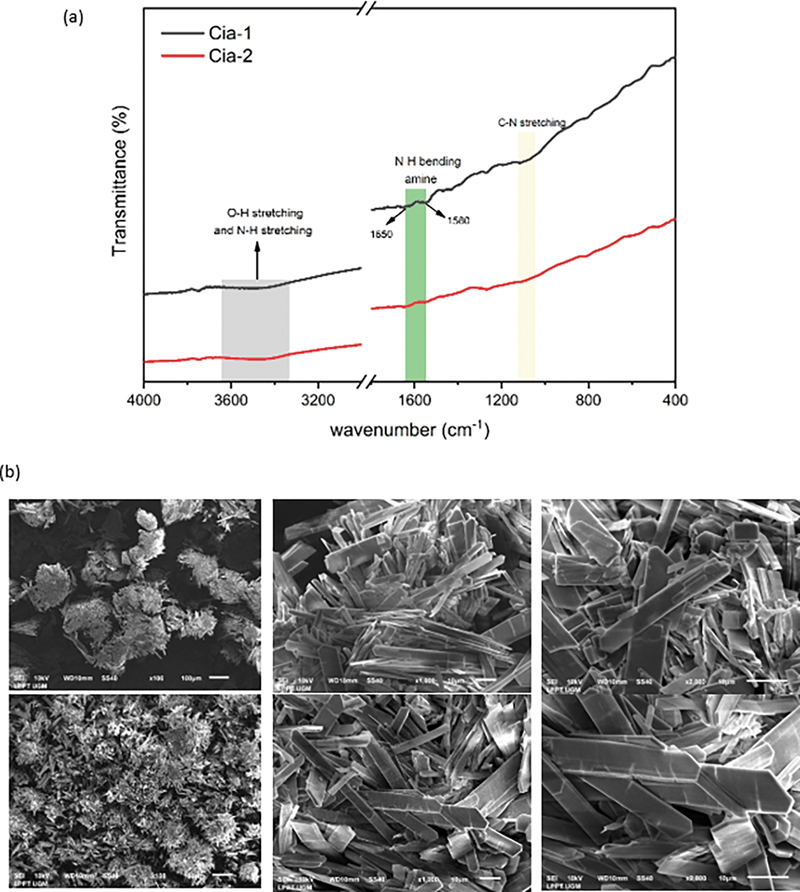
Cia material characterization—functional group spectrum (a) and particle morphology (b).

**Fig. 2:**
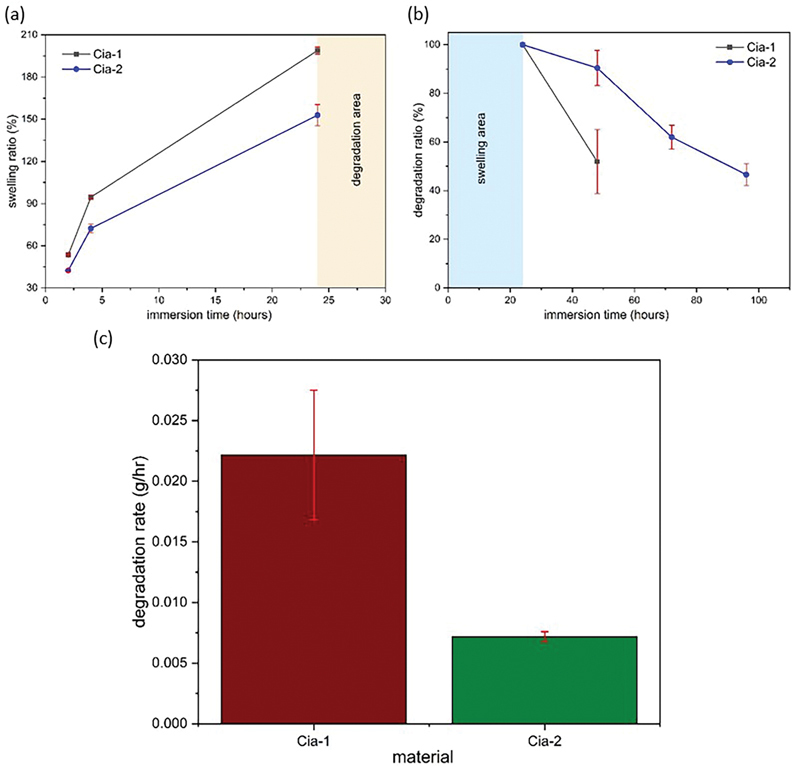
Material properties swelling ratio (a), degradation ratio (b), and degradation rate (c).

The functional groups of the synthesized Cia material were identified using the FTIR spectrum. Fig. 1a shows insignificant differences in spectra between Cia-1 and Cia-2 FTIR spectra. According to Cia-1 or Cia-2 spectra, the unsharpened peak in the wave number at 1,580 cm ^−1^ and 1,650 cm ^−1^ indicates the N-H (amine) groups from gelatin. 15 The absorption band at 1,000 to 1,100 cm ^−1^ allegedly of C-N bond from gelatin–cinnamaldehyde interaction. 18 22 Its interaction C-N between gelatin and cinnamaldehyde in Fig. 1a was indicated by Schiff bases. 16 23 Although the composition of gelatin was more dominant than others, the C = O bond which indicates of gelatin structure was not clearly seen in Fig. 1a . The particle morphology was observed using SEM. Fig. 1b shows the morphological image of Cia material indicating of crystalline shape of material in Cia-1 and Cia-2. The microstructure exhibited a crystalline appearance, with distinct rectangular formations indicative of CaCO _3_ particles. 24 The swelling behavior of the Cia materials was evaluated by immersing the samples in PBS (pH 7.4, 37°C). The results showed that Cia-1 had a significantly higher swelling ratio compared with Cia-2. Over 24 hours, the cinnamaldehyde addition of 2% in Cia-2 has a significant effect on swelling and degradation ratio, respectively, 152.84 ± 7.46% for 24 hours and 46.57 ± 4.52% for 96 hours, as shown in Fig. 2a, b . The degradation profile of the samples was monitored in SBF over 96 hours. The degradation rate of Cia-2 is 7 × 10 ^−3^ ± 0.4 × 10 ^−3^ g/h, as shown in Fig. 2c . For the additional information, each ingredient (CaSO _4_ , CaCO _3_ , gelatin, cinnamaldehyde, sodium citrate, and camphorquinone) has different solubility characteristics. Gypsum has moderate water solubility and slowly dissolves in aqueous environments, releasing calcium and sulfate ions essential for bone mineralization. The dissolution of gypsum contributes to controlled degradation in scaffold applications, ensuring gradual calcium ion availability for bone regeneration. CaCO _3_ is poorly soluble in water but can dissolve more readily in acidic environments (e.g., *in vivo* physiological conditions with local acidification due to osteoclast activity). The presence of carbonic acid (H _2_ CO _3_ ) from CO _2_ interactions enhances calcium ion release, which is critical for osteoconductivity in bone scaffolds. Gelatin dissolves when heated and forms a gel upon cooling. It provides a biocompatible and biodegradable matrix that enhances the structural integrity of the scaffold while allowing controlled degradation over time. Cinnamaldehyde is hydrophobic and has low solubility in aqueous environments, necessitating dispersion techniques within the hydrogel matrix. This limited solubility prolongs its release, ensuring sustained antibacterial activity and preventing rapid depletion. Sodium citrate acts as a stabilizing agent and chelating agent, preventing premature precipitation of calcium ions and enhancing material uniformity. As a UV-responsive photoinitiator, camphorquinone undergoes radical polymerization when exposed to UV light, enhancing cross-linking within the hydrogel scaffold. Since it is hydrophobic, its interaction with the hydrogel matrix is critical for maintaining polymerization efficiency.

## Discussion

Fig. 2a shows the swelling percentage of Cia-1 and Cia-2 when immersed in the SBF solution. The Cia-1 sample has a greater maximum swelling degree than the Cia-2 sample. The maximum difference in the swelling ratio between the two samples is around 35%. This is because the lower concentration of cinnamaldehyde makes the sample more hydrophilic. 20 Studies have shown that cinnamaldehyde can modulate the swelling ratio of hydrogel-based biomaterials by affecting the cross-linking density and the overall polymer network structure. In gypsum-hydrogel composites, cinnamaldehyde's hydrophobic nature may reduce water uptake initially, leading to a slower swelling response. 25 However, over extended immersion times, the gradual diffusion of cinnamaldehyde into the surrounding medium could modify the material's internal structure, allowing more water to penetrate, and increasing the swelling ratio. Additionally, the interaction between the gypsum particles and the hydrogel matrix can be altered by cinnamaldehyde, enhancing the mechanical integrity of the composite while controlling the rate of water absorption. 23 Time-dependent swelling studies have indicated that materials immersed for prolonged periods show a more pronounced effect, with cinnamaldehyde enhancing the equilibrium swelling ratio by modifying the network's porosity and water diffusion pathways. 26 27

The swelling ratio, a critical indicator of hydrogel functionality, is time dependent and influenced by the presence of additives such as cinnamaldehyde. 28 Immersion time plays a crucial role in determining the degradation behavior of cinnamaldehyde-infused composites, particularly those based on materials like gypsum-hydrogel systems. 29 Over time, water penetrates the composite matrix, causing changes in both the physical structure and chemical composition of the material. Cinnamaldehyde, being a bioactive and volatile compound, undergoes gradual leaching and chemical transformation when exposed to aqueous environments. 30 Initially, during short-term immersion, the hydrophobic nature of cinnamaldehyde may slow its release into the water, creating a delayed degradation response in the composite. 31 However, as immersion time increases, the water gradually permeates the hydrogel matrix, breaking down internal bonds and allowing cinnamaldehyde to diffuse out more readily, thereby accelerating the degradation process. 32 33 The impact of the higher hydrophobicity of the scaffold material with the addition of cinnamaldehyde concentration is shown in Fig. 2b . In the Cia-2 sample, the degradation time of the sample until it runs out shows up to after 96 hours or approximately three times longer than the Cia-1 sample. Protein-based scaffolds have high hydrophilicity, so when the material environment is in high water abundance, it will trigger the hydrolysis process. 28 This can be minimized by the addition of cinnamaldehyde, which is getting bigger, so that the lysis process by water will be low because the interaction of water molecules with polar compounds on the surface is getting smaller.

**Fig. 3:**
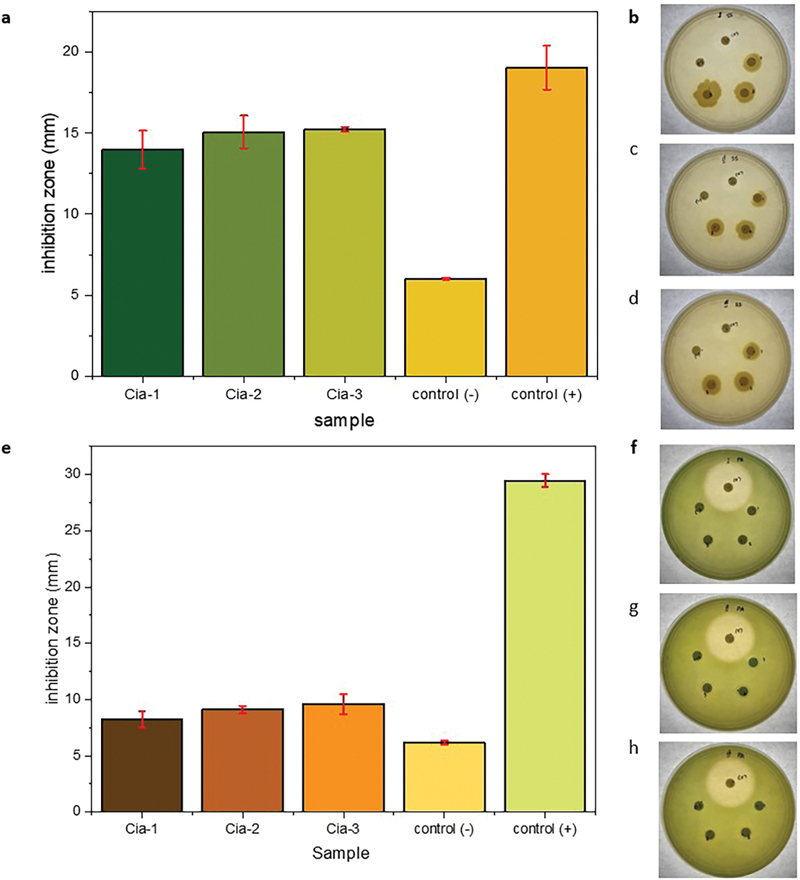
Antibacterial activity of Cia materials to *S. sanguinis* (Gr + ) (a–d), and *P. aeruginosa* (Gr − ) (e, f).

In composites where cinnamaldehyde is more strongly bound to the polymer network, the degradation is slower, with the hydrophilic–hydrophobic balance of the hydrogel influencing the release rate. 34 During overextended immersion periods, degradation may reach a plateau as most cinnamaldehyde is released, leaving behind a more porous and weakened matrix. This prolonged exposure to water also accelerates the breakdown of the hydrogel and gypsum components, further facilitating the release of any remaining cinnamaldehyde. 35 As the immersion time continues, mechanical properties such as the strength and integrity of the composite also degrade, which is exacerbated by the loss of cinnamaldehyde, indicating a strong correlation between immersion time and the degradation rate of the composite. 17 The character graphs of the degradation rate and swelling ratio between the Cia-1 and Cia-2 samples have significant differences. Cinnamaldehyde, which is hydrophobic, will cause an increase in the hydrophobicity character of a material. 36 37 The higher addition of cinnamaldehyde concentration results in the material having more nonpolar molecules, making it more difficult to interact with water molecules in the material environment. This results in the diffusion process of water molecules into the material a little, and the interaction between the compounds that make up the material and water molecules in the environment is getting lower. The impact of this process is that the swelling ratio of the material becomes lower. In addition, this has the effect that the Cia-2 material has a low degradation rate because the process of hydrolysis of the material by water is lower. Besides its characteristics to improve the hydrogel material properties, cinnamaldehyde becomes an antibacterial agent. The antibacterial activity of Cia materials is shown in Fig. 3 , the inhibition zone of bacteria. The ability to prevent bacterial growth and antibiofilm was conducted using *S. sanguinis* and *P.* *aeruginosa* . 28 Fig. 3 shows that the inhibition zone of *S. sanguinis* is larger than *P. aeruginosa* . The average inhibition zone when Cia materials are applied as antibacterial forming is within 15 mm in cinnamaldehyde 2% (Cia-2). The positive control gives an inhibition zone of around 18 mm. Cia materials indicate a significant antibacterial effect on gram-positive bacteria. 16 18 Contrasting the cinnamaldehyde effect on gram-positive bacteria, the ability to inhibit the biofilm and growth of *P. aeruginosa* is insignificant.

### *In vivo* Study

**Fig. 4:**
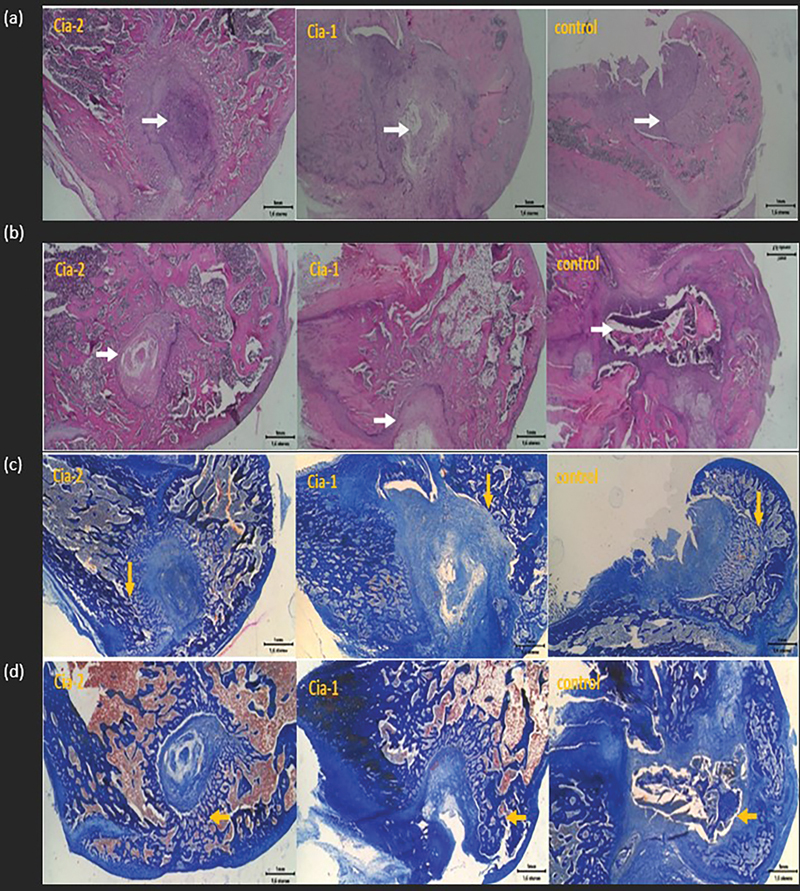
The result of implantation for 14 days (a, c) and for 30 days (c, d). Artificial defect in femoral condyle bone of 4-month-old Wistar rats. Paraffin embedding was followed by a staining process using hematoxylin–eosin (a, b) and Mallory staining (c, d).

On the 14th day of implantation, it appeared that Cia-2 showed better bone regeneration compared with Cia-1 and control (without cinnamaldehyde) in Fig. 4 . In all groups, it was shown that the bone scaffolding material still appeared, meaning it was not degraded. The scaffold material was still surrounded by inflammatory cells. New bone growth along with the degradation of scaffolding material starts from the edge of the defect to the middle. The boundary between new and old bones is clear, and by HE staining, the inflammatory cells are still visible, especially in the center of the defect (white arrow). Mallory staining shows the presence of collagen fibers that are marked with a blue color. The gradation of light blue to dark blue describes the density of collagen fibers that are increasingly compacted and form new bone around the defect. A darker blue color indicates higher density. There is a firm boundary between the old bone and the new bone in a defect (yellow arrow). New bone growth appeared to be the fastest Cia-2 and reduced to both Cia-1 and a control, respectively. Cinnamaldehyde has anti-inflammatory and antibacterial effects that help accelerate wound healing and the growth of new bone cells. On the 30th day of implantation, it appears that the best bone formation is Cia-2. Defects in Cia-2, Cia-1, and control have not closed on the 30th day of implantation. This indicates that the new bone regeneration process has not been completed and is still continuing the next day. In group B, the process runs slower than in group Cia-2. The control has the least regeneration of new bone in the defect area, and there is even an empty space in the center which indicates that the implant has been degraded before the formation of bone is complete. Around the defect grows fibrosis tissue so that recurrence is disturbed. Subcutaneous implantation is performed for 3, 7, and 14 days. It appears that on the third day, bone scaffolding material is still not much degraded in all groups. On day 7, Cia-2 seemed still the most material compared with groups Cia-1 and a control. Inflammatory cells were still scattered between the materials, marking that the wound-healing process was still taking place. Groups Cia-2, Cia-1, and a control on day 14 showed almost the same wound healing after implantation and the remaining material was getting smaller.

**Fig. 5:**
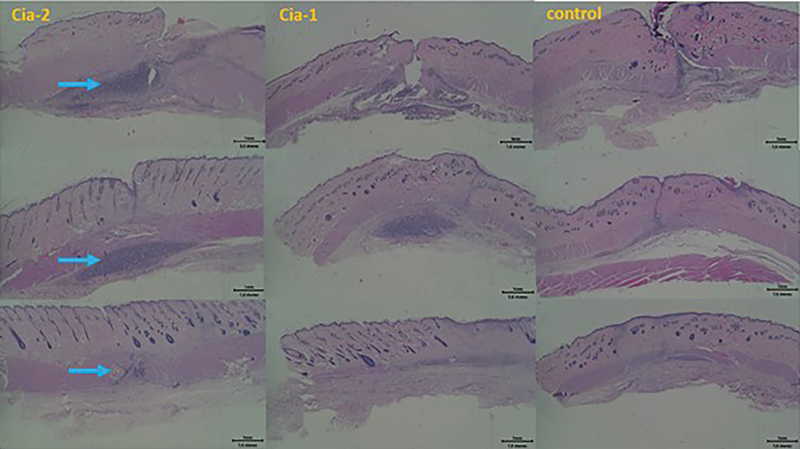
The result of subcutaneous implantation for 3 days (top), 7 days (middle), and 14 (bottom) days in 4-month-old Wistar rats. Paraffin embedding was followed by a staining process using hematoxylin–eosin.

**Fig. 6:**
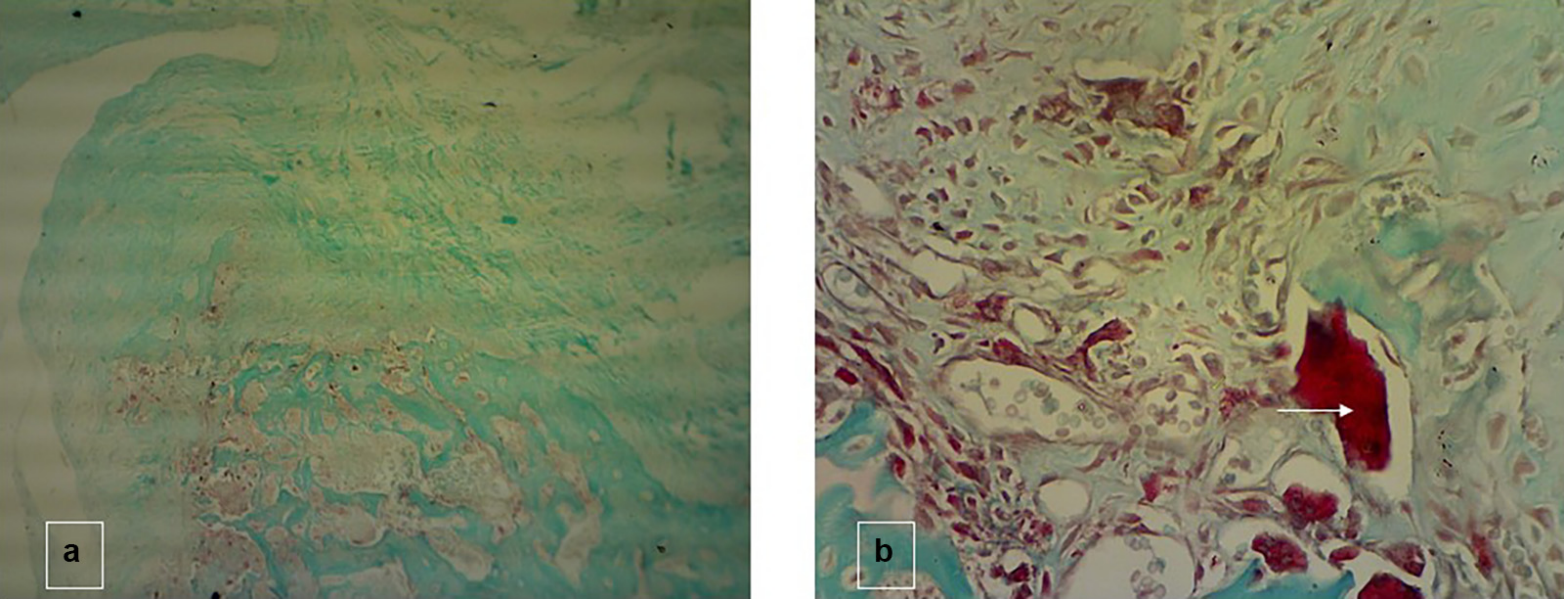
The result of implantation for 30 days for Cia-2. Sections were stained with TRAP in observation for ×40 (a) and ×400 (b) magnification. The appearance of osteoclast by TRAP staining (white arrow) indicates that the remodeling process was found in the surrounding defect area. TRAP, tartrate-resistant acid phosphatase.

A bone substitute material will be in contact with hard tissue (bone) and soft tissue (periosteum, connective tissue, and muscle). The soft tissue response to the implant sometimes shows more severe inflammation than the bone. Excessive inflammation and fibrosis in the application of biomaterials cause a failure in the healing process. 38 There are several stages of the bone regeneration process in the implantation process: First, the formation of a hematoma around the graft and after that, followed by the occurrence of tissue necrosis around the graft and attracting inflammatory cells to that area. The formation of fibrovascular stroma prepares new blood vessels and triggers osteogenesis in the graft area. Indicators of implantation success are the formation of new bone and the occurrence of a remodeling process without the formation of fibrosis tissue. 39 The healing and wound closure process should occur around soft tissue implantation. The blue arrow in Fig. 5 shows the bone scaffold material with inflammatory cells around. 40 41 Further, histological analysis revealed in Fig. 6 that there was abundant osteoclast activity inside the defect with the tartrate-resistant acid phosphatase polyclonal antibody staining all groups. The positive cells (red color) consisted of monocytes, preosteoclasts, and osteoclasts on the 30th day of implantation. The osteoclastic cells were detectable as contrast-stained cells closely attached to the newly formed bone inside the defect. 42 Cinnamaldehyde has been reported for its potential wound-healing activity, associated with its antimicrobial and anti-inflammatory effects. The greater the content of cinnamaldehyde in the formulation of bone scaffolding materials, the more it will affect the growth rate of new bone and reduce inflammation around bone defects in both hard and soft tissues. 43

## Conclusion

Cinnamaldehyde's presence could affect the polymerization of composite material while curing using UV light. Materials with the addition of greater cinnamaldehyde concentrations show better characteristics in the form of degradation rate, lower swelling ratio, and longer degradation time. The optimum concentration of cinnamaldehyde addition is 2% toward antibacterial ability. A significant antibacterial activity material effect on *S. sanguinis* bacteria. The greater the content of cinnamaldehyde in the formulation of bone scaffolding materials, it will affect the growth rate of new bone and reduce inflammation around bone defects, both hard and soft tissues.
